# Indian‐enriched genetic variants are associated with cognitive function

**DOI:** 10.1002/alz.093199

**Published:** 2025-01-03

**Authors:** Hasan Abu‐Amara, Wei Zhao, Zheng Li, Yuk Yee Leung, Gerard D. Schellenberg, Li‐San Wang, Priya Moorjani, A.B Dey, Sharmistha Dey, Xiang Zhou, Alden L. Gross, Jinkook Lee, Sharon L R Kardia, Jennifer A Smith

**Affiliations:** ^1^ University of Michigan School of Public Health, Ann Arbor, MI USA; ^2^ University of Michigan Institute for Social Research, Ann Arbor, MI USA; ^3^ Penn Neurodegeneration Genomics Center, Perelman School of Medicine, University of Pennsylvania, Philadelphia, PA USA; ^4^ Penn Neurodegeneration Genomics Center, Department of Pathology and Laboratory Medicine, University of Pennsylvania Perelman School of Medicine, Philadelphia, PA USA; ^5^ Penn Neurodegeneration Genomics Center, Dept of Pathology and Laboratory Medicine, University of Pennsylvania, Philadelphia, PA USA; ^6^ University of California, Berkeley, Berkeley, CA USA; ^7^ Department of Geriatrics, All India Institute of Medical Sciences, New Delhi India; ^8^ All India Institute of Medical Sciences, New Delhi, New Delhi India; ^9^ Johns Hopkins Bloomberg School of Public Health, Baltimore, MD USA; ^10^ University of Southern California, Los Angeles, CA USA

## Abstract

**Background:**

The prevalence of dementia in India is approximately 7.4% among those aged 60 years and older, yet little is known about genetic risk factors for dementia in this population. Examining genetic variants at higher frequency in India than other ancestries (i.e., Indian enriched variants) presents an opportunity to detect genetic effects which might be unique to the Indian population and/or underpowered for detection in other ancestral groups. We examined associations between Indian‐enriched variants and cognitive outcomes in 2680 older adults from the Longitudinal Aging Study in India ‐ Diagnostic Assessment of Dementia (LASI‐DAD) to identify potentially novel variants that may influence cognitive aging and dementia.

**Method:**

Using whole‐genome sequence (WGS) data, we identified 4.8M Indian‐enriched variants, including 4.2M rare variants (0.2%<MAF_LASI‐DAD_ < 5%, and MAF_LASI‐DAD_/MAF_EA_>5; and 600K common variants (MAF_LASI‐DAD_≥5%, and MAF_LASI‐DAD_ /MAF_EA_>3). We conducted single‐variant association tests for all Indian‐enriched variants with the Hindi Mental State Exam (HMSE) score, a general cognitive function score, and five cognitive domain scores. Given the potential for heterogeneity between sexes, we also added variant‐by‐sex interaction terms to test the joint effects of variants and variant‐by‐sex interactions. In all analyses, we controlled for age, sex, state, education, literacy, rural/urban status, the first 10 genetic principal components, and genetic relatedness.

**Result:**

At 5% false discovery rate, 11 variants were associated with one or more cognitive outcomes, including rs118075114, an intronic variant of *CA12* that is in a known risk locus for Alzheimer’s disease in European ancestry. Joint models identified an additional 17 variants with a sex‐specific association with HMSE score (11 in females and 6 in males). Although the identified variants are rarely reported in the literature, partly because they are almost absent in European populations and elsewhere, several of the loci have been associated with neuropsychiatric traits and dementia risk factors such as diabetes, blood pressure, and coronary artery disease.

**Conclusion:**

These results demonstrate the potential of LASI‐DAD WGS to identify Indian‐enriched variants that are associated with cognition at older ages. Future studies are warranted to replicate and understand the functional relevance of the identified variants.

